# Sweet Taste Receptor Signaling Network: Possible Implication for Cognitive Functioning

**DOI:** 10.1155/2015/606479

**Published:** 2015-01-11

**Authors:** Menizibeya O. Welcome, Nikos E. Mastorakis, Vladimir A. Pereverzev

**Affiliations:** ^1^World Scientific and Engineering Academy and Society, Ag. Ioannou Theologou 17-23, Zografou, 15773 Athens, Greece; ^2^Department of Industrial Engineering, Technical University of Sofia, 8 Kl. Ohridski Boulevard, 1000 Sofia, Bulgaria; ^3^Department of Normal Physiology, Belarusian State Medical University, Dzerzhinsky Avenue 83, 220116 Minsk, Belarus

## Abstract

Sweet taste receptors are transmembrane protein network specialized in the transmission of information from special “sweet” molecules into the intracellular domain. These receptors can sense the taste of a range of molecules and transmit the information downstream to several acceptors, modulate cell specific functions and metabolism, and mediate cell-to-cell coupling through paracrine mechanism. Recent reports indicate that sweet taste receptors are widely distributed in the body and serves specific function relative to their localization. Due to their pleiotropic signaling properties and multisubstrate ligand affinity, sweet taste receptors are able to cooperatively bind multiple substances and mediate signaling by other receptors. Based on increasing evidence about the role of these receptors in the initiation and control of absorption and metabolism, and the pivotal role of metabolic (glucose) regulation in the central nervous system functioning, we propose a possible implication of sweet taste receptor signaling in modulating cognitive functioning.

## 1. Introduction

Taste receptors are integral plasma membrane proteins that recognize sapid substances, code information received from these substances, and transmit the information into intracellular acceptors. Taste receptors are divided into two types: type 1 receptor recognizes sweet molecules (see examples below); type 2 recognizes bitter molecules such as toxins, acids, and alkaloids. Both receptor types were only recently characterized [1, 2] and are increasingly studied in recent time. Type 1 receptor is further subdivided into three subtypes (T1R1, T1R2, and T1R3). For type 2 receptor, at least 25 subtypes are known to exist in humans [1, 3, 4].

This paper deals only with the signaling network of the sweet taste receptors, precisely the role of their signaling network in cognitive functioning. Sweet taste receptor signaling network is a complex communication pattern involving the regulated signaling of sweet molecules activating downstream target of taste cells and resulting in the perception of taste as well as modulation of related signaling pathways. The network involves the activating substrate, sweet taste receptor, intracellular molecules and cooperatively associated receptors, secretory peptides, molecules, and ions. It is suggested that through these components, sweet taste receptors modulate paracrine signaling pathways and can significantly affect neighboring cells by changes in ion (calcium) waves and activity-dependent signaling.

The activating ligands of sweet taste receptor are diverse and include both artificial (acesulfame potassium, aspartame, neotame, sucralose saccharin, or glycyrrhizin) and natural (glucose, lactose, fructose, galactose, maltose, and sucrose; amino acids including glycine, alanine, threonine, D-tryptophan, and D-histidine; the dipeptide L-aspartyl-L-phenylalanine and sweet proteins such as monellin, thaumatin, and brazzein) sweet substances [5–7]. Functional forms of the sweet taste receptor subtypes are known to exist in dimers. For instance, T1R2 forms a dimer with T1R3 (T1R2+T1R3 heterodimer). Formation of dimers and complexes allows the sweet taste receptors to detect various types of taste [4].

Sweet taste receptors have multisystem localization. The existence of sweet taste receptors was initially proposed by Newson et al. (1982) and later discovered in the gastrointestinal tract [8, 9] and then in the pancreas [7, 10–15]. They are also present in macrophages [16] and respiratory track, where it is believed to play significant role in the maintenance of the mucosal and ciliary functioning, in part, by ensuring adequate and supportive role for the sensing of tasty substances, as well as the clearance of glucose through GLUT 1 and GLUT 10 receptor types present in the respiratory track [17]. The supportive role of sweet taste receptors to glucose absorption and metabolism is proposed to play a part in the gastrointestinal tract [18], and this role probably is mediated through paracrine signaling or cross-talks [19]. These receptors are known to play a vital role in the initiation and progression of pathological process in the respiratory track (inflammation, asthma, etc.), gastrointestinal tract, and pancreas (metabolic disease such as diabetes) [7, 14, 15].

Interestingly, sweet taste receptors have been discovered in the visual, auditory, and olfactory systems, where they are known to modulate taste through visual, auditory, and olfactory perception, respectively [20–23].

Researchers have shown that sweet taste receptors are also located in the central nervous system (CNS), precisely in the hypothalamus. Ren and colleagues [24] showed that sweet taste receptor T1R2+T1R3 heterodimer is responsible for sensing glucose in the hypothalamus. This discovery could have implication for a better understanding of brain functioning and could provide information on mechanism of CNS disorders in which dysregulation of metabolism (glucose) is observed [25, 26].

The continuous search for different treatment options of cognitive disorders or the prevention of such conditions provides a substantial argument for constantly rising prevalence of CNS disorders in the world. Over the past decades, there has been constant increase in the prevalence of CNS disorders. It has been estimated that the number of people suffering from CNS disorders will get a whooping increase by 2020. Millions of people are mentally disabled, with the highest proportion occurring in ages 10–29 years [25]. Only between 1990 and 2010, the burden of disorders associated with CNS increased by 37.6%. In 2011, it was reported that neuropsychiatric disorders (accounting for 45%) were the leading cause of disability for people aged 10–24 years [25, 27]. These data suggest that, indeed, there is increasing necessity to search for new frontiers in both metabolic and cognitive functioning of the CNS. This is based on increasing evidences suggesting that metabolic disorders precede cognitive dysfunction [28]. Cerebral metabolic regulation is key to normal cognitive functioning and might in fact be a key predictor of cognitive functioning and diseases related to brain functioning. In fact, in an analysis, it was observed that cerebral glucose regulation parameter for the identification of cognitive dysfunction was more effective and efficient than the neuropsychological tests that are used for the diagnosis of cognitive impairment [28]. For instance, researchers have shown that the initial stage of Alzheimer's disease involves decrease in brain glucose metabolism by 45%, whereas blood flow decrease by only ~18% [29]. Assuming conservatively that the prevalence of the disease remains constant, it is estimated that for Alzheimer's disease alone, compared with the prevalence as at 2010, the number of cases will double, hitting 65.7 million by 2030 and 115.4 by 2050 [30]. It is estimated that the sharpest increases in the disease are expected to hit low and middle income countries [31]. A huge amount of economic, societal, and psychosocial costs accrue from cognitive impairments [32–34]. In 2010, the total worldwide cost of cognitive impairment was estimated at US$604 billion [31]. In the US alone, an estimated cost of caregiving in 2012 was at $216 billion [35], suggesting a sharp increase in the economic cost due to the increase in the prevalence of the disease. A total of $536 billion and $1.75 trillion are minimum estimates of the long-term losses to the US economy in 1991 caused by Alzheimer's disease [36]. Without doubt, it is obvious that understanding how the sweet taste receptors could affect metabolic and cognitive functions of both neurons and astrocytes could provide plausible information on treatment option of some CNS disorders that have recorded tremendous increase in rent times. Of paramount importance is how efficient hypothalamic metabolic regulation relates to the regulation of metabolism in other parts of the brain (such as the hippocampus and cortex).

Based on data that suggest possible role of the sweet taste receptors in controlling sugar absorption and metabolism and recent reports that metabolic disorders precede cognitive impairment, it is suggested in this work that the activity of this sweet taste receptor-signaling network could have implication for the regulation of some aspects of cognitive functioning. In this paper, recent studies that suggest a possible role of the sweet taste receptor in the neural control of metabolism and cognition are reviewed. The possible mechanisms linking the cognitive and metabolic functions of the sweet taste receptors are also proposed.

## 2. General Model of Signaling of Sweet Taste Receptors

**Figure 1:**
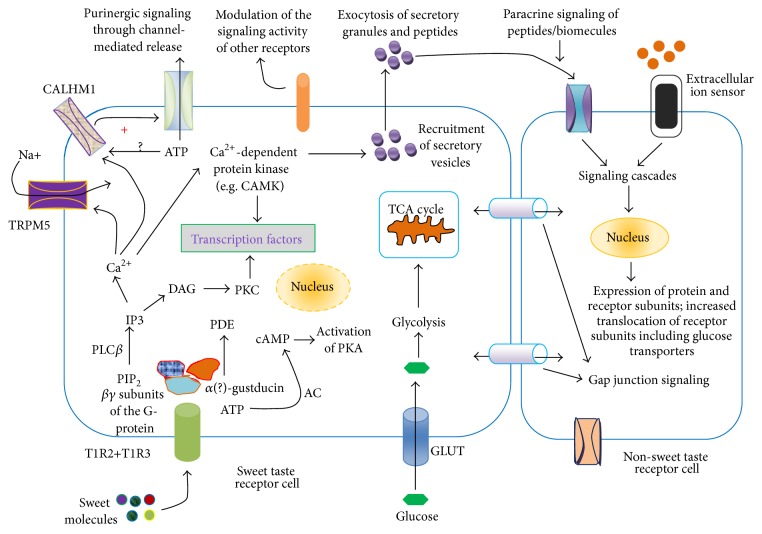
A general model of sweet taste signaling network. Sweet taste receptors possess multiple binding sites and mode of interaction for sweet molecules and they belong to class C of heterotrimeric guanine nucleotide-binding protein, G-protein [143–145]. Sweet molecules activate the G-protein by downstream signaling leading to the dissociation of the *α*-subunit gustducin from the *βγ* subunits [146, 147]. Dissociated *βγ* subunits of the G-protein activate phospholipase C*β* (PLC*β*), leading to the formation of 1,4,5-inositol trisphosphate (IP3). IP3 is responsible for the release of Ca^2+^ from intracellular stores through its binding to IP3-receptor in these stores. Increase in intracellular Ca^2+^ activates calcium dependent kinase, monovalent selective cation channel, TRPM5 (transient receptor potential cation channel, subfamily M, member 5) [15, 44, 146], and other receptors [44, 148]. To establish the role of TRPM5 or PLC*β* (type 2), Zhang et al. [4] showed that knockout of the receptor or the enzyme abolishes the sensation of taste in cells. TRPM5 may also play a role in capacitance mediated calcium entry into taste cells [147]. Modulation of purinergic signaling by taste receptor also plays useful role in taste sensation. For the initiation of purinergic release, it was recently demonstrated by Taruno et al. [148] that the voltage-gated ion channel, calcium homeostasis modulator 1 (CALHM1), is indispensable for taste-stimuli-evoked ATP release from sweet, bitter, and umami taste cells. Importantly, CALHM1 is expressed not only in sweet but also in bitter and umami taste sensing type 2 cells. Taruno et al. [148] proposed that CALHM1 is a voltage-gated ATP-release channel. Dissociated *α* subunit referred to as G*α*-gustducin activates a phosphodiesterase (PDE) thereby decreasing intracellular cAMP levels [146, 149]. G*α*-gustducin is also reported to activate adenylate cyclase (AC) to increase cAMP level [150]. According to earlier report, Clapp et al. [151] demonstrated that, compared to wild type mice, knockout of *α*-gustducin in the taste buds of mice resulted in high basal (unstimulated) cAMP level. The results of these authors [151] indicated that *α*-gustducin is necessary to maintain low level of cAMP level. Low level of cAMP is necessary to maintain the adequate signaling of Ca^2+^ by disinhibition of cyclic nucleotide-inhibited channels to elevate intracellular Ca^2+^ [38]. Changes in cAMP levels also affect other ion channels, including K^+^ channels. The events resulting in activation/modulation of ion channels lead to membrane depolarization and formation of action potentials. Potential-dependent release of mediators (ATP, serotonin, etc.) and peptides and calcium dependent release of peptides/biomolecules are some of the results of sweet taste receptor signaling [152]. A hallmark of sweet taste receptor signaling is the activation of transcription factors and gene expression, which might be dependent on calcium and activity dependent activation calcium dependent kinases, including the calmodulin-dependent protein kinase (CAMK). Activation of protein kinases may be achieved through other signaling pathways. It appears that sweet taste receptor signaling involves multiple activating substrates and different types and subtypes of both *α*-gustducin and *βγ* subunits of the G-protein. Although, different subtypes of sweet taste G-protein receptor subunits have been known for over a decade, their specific roles in sensing taste are not exactly clear [38, 149, 153]. For instance, Huangu et al. [149] reported the presence of *β*1 and *γ*13. The sweet taste receptor is also known to have *β*3 subtype subunit. For *α*-gustducin, G*α*_i-2_, G*α*_i-3_, *Gα*_14_, *Gα*_15_, G*α*_q_, G*α*_s_, *α*-transducin have been identified [38].

It is at least 3 decades since the initial hypothesis about taste receptors was made in early 1980s by Newson and colleagues [37]. However, experimental results on the presence of these receptors showed up in the literature only after a decade following the proposal of Newson and colleagues [8, 9]. Literature data point to the extensive development of sweet taste receptor signaling in the last half-decade. This development involved not only the unraveling of some of the mechanisms of sweet taste receptor signaling but also the diversity in the localization. The discovery of sweet taste receptors in the brain is a key to better understanding of certain aspect of brain functioning. Ren et al. [24] reported the localization of sweet taste receptors in the brain and suggested that these receptors serve as glucosensor in the hypothalamus. Signaling mechanisms of sweet taste receptors in the identified tissues and cells bear some similarities. In this next section, a general model of sweet taste receptor signaling will be outlined; thereafter, the role and mechanisms of these receptors in metabolic and cognitive functions shall be discussed. The general concept of sweet taste receptor signaling is shown in Figure 1.

The model (Figure 1) shows the mechanism of signaling initiated by sweet substances in sweet taste cell and possible effect on neighboring nonsweet taste cell (through paracrine or gap junction communication). One aspect of research into sweet taste receptor signaling that is yet to be reported is how different concentrations of sweet substances affect signaling.

The signaling events (Figure 1) lead to the modulation of cellular activities, propagation of action potential, and the paracrine signaling [38–40], including the release of peptides (glucagon-like peptide-1, GLP-1) and several biomolecules [41]. The functions of GLP-1 are well documented in the brain and gastrointestinal system [42].

Increasing evidences suggest that sweet taste signaling mechanisms in the gastrointestinal tract are similar to those in the pancreas and respiratory tract [19]. Nakagawa et al. [43] showed that sweet taste receptors of the pancreatic *β*-cell (by the addition of sucralose) activate the calcium and cAMP signaling systems to stimulate insulin secretion. They demonstrated that the secretory activity of the second pool of insulin vesicles blocked by the dihydropyridine L-type calcium channel blocker, 3,5-dimethyl 2,6-dimethyl-4-(2-nitrophenyl)-1,4-dihydropyridine-3,5-dicarboxylate (nifedipine). In addition, the IP3-receptor inhibitor, 2-aminoethoxydiphenyl borate, effectively blocked Ca^2+^ waves caused by both the first and the second pool of insulin secretion. More so, the peptide, gurmarin, an inhibitor of the sweet taste receptor, blocked calcium response to the artificial sweetener, sucralose [43]. Gurmarin can inhibit sweet taste responses 30–80% to multiple sweet molecules, including glucose, sucrose, saccharin, and SC45647; however, it has no inhibitory effect on salty, sour, or bitter compounds. Another known inhibitor of sweet taste receptor includes the proteolytic enzyme pronase [44]. These results are in agreement with the reports of [45] and data reported elsewhere [46] for other tissues and cells. The heterodimer sweet taste receptor, T1R2/T1R3, in the presence of amiloride (3 mM) loses its response to sweet taste molecules such as sucrose, artificial sweetener, and sweet protein. Another sweet taste inhibitor, lactisole, is known to inhibit this response but maybe these inhibitors may possess different binding sites on the sweet taste receptor dimers [45].

The presence of multiple sweet taste signaling pathways is based on experiments suggesting that gurmarin inhibits some but not all sweet taste responses of the chorda tympani nerve to sweet molecules in experimental animal [44]. Investigators showed that gurmarin inhibition of sweet taste receptors was dependent on many factors, including temperature, suggesting that multiple factors, including peptides, environmental factors, and higher brain functions (such as emotion) may play a role in modulating taste perception. Yoshida et al. [47] recently demonstrated that the anorexigenic mediator and adipocyte hormone, leptin selectively suppresses sweet taste sensitivity via the adiposity receptor, Ob-Rb, in sweet taste cells. Whereas orexigenic mediators, endocannabinoids, notably, anandamide [N-arachidonoylethanolamine], and 2-arachidonoyl glycerol, selectively enhance sweet taste via cannabinoid receptor type 1 in sweet taste cells. These mediators act centrally in the hypothalamus and limbic forebrain [47]. While this present work did not find any study investigating the effect of insulin on sweet taste receptors, it could be expected that increase in insulin secretion signaling will lead to suppression of sweet taste, while glucagon will enhance sweet taste perception in sweet taste cells.

## 3. The Sweet Taste Receptor Heterodimer, T1R2+T1R3, as a Model Sensor of Glucose in the Neuroastroglial System: Window of Interaction with Cognitive Control Systems

Generally, sensors of glucose could be divided into plasma membrane glucosensors and intracellular glucosensors. The functions of these different types of glucosensors will be discussed in more details in another paper under preparation. In this section, only the plasma membrane glucosensor, T1R2+T1R3, is discussed, precisely, as a hypothalamic glucosensor serving to modulate metabolic and cognitive functions in the neuroastroglial system. However, it is important to note that there are numerous interactions between the plasma membrane and intracellular glucosensors, which will be briefly outlined in course of our discussion. At present, in the world literature, known plasma membrane receptors that serve as glucosensors are the second member of the glucose transporter (GLUT2) and the third member of sodium/glucose cotransporter, SGLT3. The heterodimer, T1R2+T1R3, is a fairly new member of the plasma membrane glucosensors.

As earlier noted, numerous sweet molecules can activate the T1R2+T1R3; however, because our discussion in this section is directed to the neuroastroglial system, we shall limit the ligand of sweet taste receptor to glucose molecule alone. This is because glucose is the chief metabolic substrate for the brain. The daily requirement of glucose for the brain of an adult is about 120 g of the total amount of 160 g of glucose needed daily for the whole organism [46, 48]. During brain activation (as in mental activities), glucose uptake by brain cells can be increased up to ~90%. Our previous analysis also showed that the contribution of blood glucose level necessary to maintain the function of the brain is about 40% and may increase to 90% or more during prolonged mental work [49–51]. During hypoglycemia, brain function is greatly reduced, but ketone bodies serve (especially during continuing and prolonged fasting) to provide a significant part of the energy needs of the brain [52, 53]. However, it should be noted that ketone bodies are not able to maintain or restore the normal function of the brain in the absence of glucose [46]. In our previous analysis, decrease in glucose level was associated with statistically significant decrease in brain functions [54]. The lowering of brain functions following decrease in blood glucose level, accompanied by a corresponding decrease in cerebral glucose level is reported by McNay and Sherwin [55] and reviewed by McNay and Cotero [56] and also documented elsewhere [57–59].

The mechanisms involved in the role of sweet taste signaling [24, 60–63] in cognitive functions may largely involve signaling through metabolic coupling; activity dependent signaling; cross-signaling, initiated by downstream effectors; and receptor cooperativity and associativity. These processes may be mediated through paracrine signaling, activities of extracellular ion sensors, and ion homeostasis, which in turn may modulate the functions of the neuroastroglial system.

Ren et al. [24] found sweet taste receptors (T1R1,2,3 and their heterodimers: T1R2/T1R3 and T1R1/T1R3) as well as their corresponding G-protein genes in the neurons of the nuclei paraventricularis and arcuatus of the hypothalamus, CA area and dentate gyrus of the hippocampus, habenula, cortex, and intraventricular epithelial cells of the choroid plexus. Interestingly, the highest level of taste-related G-protein gene expression was found in the hypothalamus, followed by cortex and hippocampus [24]. The hypothalamus has been long known to serve as a site of regulation of feeding, central and peripheral metabolism, hormones secretion, and functions [24]. Expression of these receptors is associated with a physiological condition (fasting increases the amount of T1R1 and T1R2 decrease as hypothalamic glucose leads to an increase in the expression of T1R1, T1R2, which normalizes when sweet molecules were administered). Moreover, these brain areas identified to express taste receptor genes are implicated in cognitive functioning [64–66]. It is possible that receptor cooperativity or associativity with the sweet taste receptor could as well affect signaling in other pathways [67].

The chief signaling relationship between the activities of plasma membrane glucose sensor (which is also linked to intracellular energy sensors) and cognitive control systems is made possible, majorly, through metabolic coupling in the neuroastroglial system [61]. Importantly, metabolic coupling could have profound effect on activity dependent signaling; cross-signaling, initiated by downstream effectors; and receptor cooperativity and associativity by majorly modulating paracrine signaling, activities of extracellular ion sensors, and ion homeostasis [18, 24, 67].

### 3.1. Signaling through Metabolic Coupling: A Window of Interaction between Cognition and Metabolic Functions

The activity of the neural plasma membrane glucosensor, T1R2+T1R3, from the work of Ren et al. [24] is evident that the heterodimer largely contributes to maintaining the activity of the cell by controlling glucose transport. This is possibly achieved by the associativity and/or cooperativity between the GLUT2 and T1R2+T1R3. How the T1R2+T1R3 receptor affects the glucose transporters is not known, but it may be suggested that the signaling mechanisms might involve downstream effectors that subsequently modulate both the transport activity and the expression of the GLUT2 sensor. In the gastrointestinal tract, for instance, T1R2+T1R3 receptor has been shown to influence the activity and expression of GLUT2 and SGLT1, possibly through autoparacrine signaling [18]. Moreover, Margolskee et al. [18] reported that this receptor heterodimer controls glucose absorption and metabolism.

Decrease in metabolic functions of the neurons results in decrease in neuronal activity. Thus, collateral neuronal and glial cell functions might also be affected. Shunts controlled by metabolic coupling such as the GABA/glutamate-glutamine cycle will also be affected since glucose serves as precursor [68–72]. Several neurotransmitter systems implicated in cognitive functioning are regulated by glucose or its metabolites [73, 74]. Analysis of the literature indicates a significant role of dopamine, glutamate, serotonin, cholinergic, and noradrenergic systems in cognitive functioning [61, 75]. Glucose is required for the synthesis of neurotransmitters such as serotonin, noradrenaline, and acetylcholine, which can affect both local and distant neural and astroglial population [61]. The neurotransmitters, ATP, d-serine, which may be synthesized from glucose, affect long-term potentiation (LTP), synaptic plasticity, information storage, and retrieval [75, 76]. Substances released from the neuroastroglial system can also initiate signaling in many pathways necessary for memory/cognitive functions [75, 76].

The release of these molecules could modulate not only cognitive functions but also different aspects of behavior, including emotion [75–77]. The functional link between the hypothalamus and brain areas of emotion is an indication of the possible influence of hypothalamus on cognitive functioning. Moreover, the hypothalamus itself is implicated in cognitive functioning as disorders involving the hypothalamus evidently result in cognitive dysfunction [78–83] (briefly discussed in the next section).

Downstream effects of T1R2+T1R3 might also involve energy sensors. The discovery of the presence of functional sweet taste receptors in the brain has thrown more light to our understanding of metabolic functioning involving glucose regulation. Metabolic or energy sensors in the neuroastroglial system include glucokinase, GLUT2 [84, 85], AMPK (AMP activated protein kinase), CREB (cAMP related element binding protein), mTOR (mammalian target of rapamycin), sirtuins, and PASK (or PASKIN and PSK, a kinase protein) [64, 86–90]. The study by Ren et al. [24] has shown that the sweet taste receptor heterodimer T1R2+T1R3 might be responsible for mediating the energy sensing activity of previously identified metabolic sensors. Although in Ren et al. [24] study the activities of other metabolic sensors were not put into consideration, knockout of sweet taste receptors may have considerable impact on intracellular energy sensors in the neuroastroglial network. However, it will be expected that decrease in glucose entry into the cell caused by disorder in sweet taste receptor signaling will actually mobilize intracellular substrates to counterbalance the change. Notwithstanding, this change will result in decrease in neural functions due to lowering of glucose entry into the cell.

There could be a functional association between the identified metabolic sensors and plasma membrane glucosensors. Recent study by Hurtado-Carneiro et al. [91] has shown that PASK that functions as a nutrient and energy sensor in hypothalamus is required for the normal functioning of other metabolic sensors, including AMPK and mTOR/S6K1. This functional relationship (or cross-talk) between metabolic sensors is useful in maintaining not only metabolic functions but also normal/adequate cognition. In this regard, previous data have consistently shown that AMPK, CREB, and mTOR are involved in both glucose metabolism and memory functions (reviewed in [61]) (both AMPK and CREB are strongly involved in T1R2+T1R3 signaling). Recent work by Hurtado-Carneiro et al. [92] indicates that the peptide GLP-1 can attenuate the activity of AMPK and mTOR/S6 kinase induced by fluctuations in glucose levels in hypothalamic areas involved in feeding behaviour. The functional relationship between some of these signaling pathways was analyzed in a recent review [93]. Disorders involving the mTOR significantly affect cognition and is associated with neuropsychiatric symptoms, including intellectual disability, specific neuropsychological deficits, autism, other behavioral disorders, and epilepsy [94].

Although, information is scanty, available data suggest that other signaling pathways that link glucose metabolism to cognitive functioning might include extracellular kinases [95–97]. These identified pathways could activate LTP, thereby enhancing memory formation or retrieval [98, 99] and can also function through activity-dependent signaling as well as activation of transcription factors [3, 11, 100]. Decrease in glucose is associated with deficits in memory and learning (possibly due to a decrease in LTP) [101, 102].

In the gastrointestinal tract, for instance, endocannabinoid receptors are reported to be associated with the fatty acid receptor necessary for sweet taste perception [67]. In the nervous system, the literature search in this present study did not produce such result; it can, however, be proposed that such associativity is possible. In this vein, it is demonstrated that the effects of cannabinoids are associated with dysfunctions of rapamycin pathway and extracellular signal-regulated kinases, suggesting a relationship between the signaling pathways, and possibly receptor cooperativity or associativity [103]. In Wang and Zhuo's paper [104], it was noted that stimulation of group I metabotropic glutamate receptors initiated a wide variety of signaling pathways that regulate gene expression at both the translational and transcriptional levels and induce translation or transcription-dependent synaptic plastic changes in neurons. This wide range of signaling by metabotropic glutamate receptors can activate other receptors and thus initiating the activity of numerous transcription factors and gene expression.

Of important note is the downstream effect of neuroastroglial glucose metabolic disorder resulting in changes in calcium ion concentration. The result of this is a disorder in synapse-to-nucleus communication involving several kinases (including the mitogen-activating protein kinase, MAPK; CAMK), gene expression, and synaptic plasticity [105, 106]. These protein kinases are responsible for regulating the activities of transcription factors including CREB, CCAAT (cytosine-cytosine-adenosine-adenosine-thymidine)/enhancer-binding protein (C/EBP), Early growth response protein (Egr) also known as zinc finger protein 225 (Zif268) or nerve growth factor-induced protein A (NGFI-A), activator protein-1 (AP-1), nuclear factor *κ*B (NF-*κ*B), c-Fos, and Elk-1, c-Jun. These proteins (transcription factors and kinases) are well implicated in metabolism and cognition [104–110]. LTP is dependent on the activities of protein kinases. Also, nitric oxide, which is thought to be associated with glucose metabolism [111, 112], also contributes to LTP by downstream targets, stimulating guanylyl cyclase and cGMP-dependent protein kinase, which acts in parallel with PKA to increase phosphorylation of the transcription factor CREB [106]. Most notably, activity-dependent signaling of calcium is also shown to play a pivotal role in neural and synaptic plasticity and regulation of gene expression [106].

It has been proven again and again that memory formation and retrieval involve short-term signaling associated with activation of transcription factors controlling immediate early genes (early response genes) and long-term signaling to the nucleus for the formation of long-term memory [113–117]. However, the fact that microtubules of cells might play a pivotal role in memory has only been explored recently. In the work of Craddock et al. [118], a possible encoding of memory in the microtubule lattices mediated by the phosphorylation of type II CaMK was reported. Whether extended research into mechanisms of memory coding in the microtubule lattices will help scientists find the long-time searched neural code for memory is yet unknown. In the work of Eric Kandel, who shared the 2000 Nobel Prize in Physiology or Medicine with Arvid Carlsson and Paul Greengard, best known for his research on the physiological basis of memory storage in neurons, documented in his Nobel Lecture, published in the 2001 issue of the “Bioscience Reports,” there are compelling evidences for the role CAMK in memory [113]. In page 597 of Eric Kandel's account of the mechanisms of memory storage, CAMK was noted to play a role in LTP [113]. In Craddock et al. report [118], activity dependent flux of postsynaptic calcium activated the dodecameric holoenzyme containing two hexagonal sets of 6 kinase domains, hexagonal CaMK type II. One bit of information encoded equals one protein kinase domain. Information conveyed through activity dependent calcium waves are encoded by the phosphorylation as arrays of binary “bits.” Six phosphorylated bits are equal to bytes. Thus, thousands of bytes of information can be encoded in one microtubule [118]. A number of studies have implicated the role of microtubules [119–121] and CaMK signaling in both normal cognitive functioning and diseases [122, 123].

Recent work suggests the mechanism for the involvement of microtubule network in the formation of memory [124]. Wang and Zhuo [104] recently showed that microtubule protein is regulated by activity dependent processes. The mechanism of its regulation indicate that synaptic changes are associated with activation of the corresponding gene (more precisely called immediate early gene) and gene expression. The early responsegene c-fos is also involved in activity dependent signaling and it is dependent on CREB activity [104]. Also, microtubule network is previously known to interact with nuclear materials necessary for the formation of memory [125]. The turnover rate of microtubule also correlates well with time needed for short-term and long-term memory formation. It is obvious that there are millions of microtubule proteins to which protein kinase (e.g., CAMK) can interact with; however, the approximate number is not known precisely as there are numerous disagreements in the literature as to the number of microtubule proteins present in a given neuron [126, 127].

### 3.2. The Relationship between Cognition and Hypothalamic Metabolic Functions: The Search Continues

Cognition (from Latin* cognotio*, to know, learn) is a higher brain function and comprises attention, memory, judgement, reasoning, problem solving, planning, decision-making, and language. It is related to emotion and behavior. Cognition is a function of multiple brain regions, majorly involving the cortical Brodmann areas, insula, anterior cingulate, thalamus, mediotemporal (including hippocampus), mediofrontal and prefrontal cortices, basal ganglia, and cerebellum [64–66, 128–133]. The hypothalamus plays a role in some aspect of cognition, mostly emotion and behavior [134–136]. The mammillary nuclei and medial nuclei of the posterior hypothalamus are involved in some aspects of cognitive functioning. Medial part of the tuberal hypothalamus (tuberal hypothalamus is at the level of the tuber cinereum, which is usually divided into medial and lateral parts) containing the arcuate nucleus is involved in feeding and dopamine release. Dopamine can act locally and at distant point away from the site of release to modulate cognition [77].

How will disruption of sweet taste receptor signaling affect cognitive functions? Hypothalamic dysfunction is involved in epilepsy, a condition characterized by higher brain function impairment, including cognitive dysfunction [137]. In addition, the hormones and peptides secreted by the hypothalamus are involved in cognition. The secretions of the hypothalamus mediated by metabolic activities could extend to other brain areas including the hippocampus and cortex and are believed to modulate various behavioral parameters: learning and memory, as well as neuroprotection, reproduction, growth, and metabolism [138]. For instance, investigation involving the growth hormone has been shown to modulate synaptic plasticity, thereby altering cognition. Growth hormone replacement therapy attenuates cognitive impairment [139]. Apart from the growth hormone, other hormones of the hypothalamus have been implicated in cognitive functioning. Importantly these hormones of the hypothalamopituitary axis are known to act on other brain areas involved in cognitive functioning: hippocampus and cerebral cortex [78–83]. Some of these hormones can modulate taste perception [140]. These hypothalamopituitary hormones have been implicated in cognitive dysfunction, including dementia. In progressive neurodegenerative diseases involving cognitive impairment, as in Alzheimer's disease, recent evidences have pointed to the involvement of metabolic disorders as the most reliable indicator in comparison with the traditional neuropsychological tests [29, 141]. In Alzheimer's disease, alterations in hypothalamic aminergic cholinergic system is reported [141]. In Alzheimer's disease, dysfunction of the transport activity of glucose transporters have been implicated in the pathogenesis of the disease, and in metabolic disease, including diabetes (reviewed in Shah et al. [142]). These data suggest that hypothalamic metabolic alteration will affect cognitive functioning through multiple mechanisms.

## 4. Conclusion

Based on the recent finding presented in this work, the hypothalamic glucosensor, heterodimer sweet taste receptor, could serve as a key controller of glucose absorption and metabolism in the brain. This newly novel neural glucosensor will provide further opportunities for translational research aimed at identifying new therapeutic target agents to treat certain related metabolic dysfunctions of central origin and cognitive impairment, which almost in all cases coexist with neural metabolic dysfunction. Research into the polymorphic forms of the sweet taste receptors (T1R2 and T1R3), genetic variations of *α*-gustducin and their relationship to sweet sensitivity will provide useful information, including individual differences in taste identification. Investigation of the cognitive function implication of metabolic signaling functions of key brain areas involved in metabolism and cognition (hypothalamus, cortex, and hippocampus) and sweet taste receptors signaling will likely provide further information that might help in improved treatment options for cognitive impairment or provide possible cues to prevention of such conditions.

## Figures and Tables

**Figure 2:**
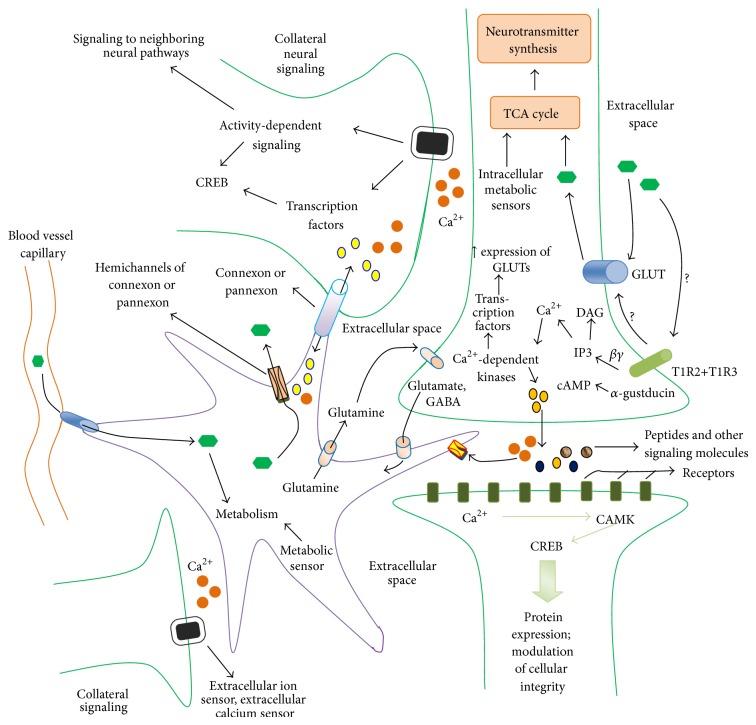
Sweet taste signaling network of the neuroastroglial system. The brain is a complex network of cells, largely populated by neurons and astrocytes. There are ~100 billion neurons, with glial cells outnumbering neurons by about 10-fold. Astrocytes form the largest population of glial cells. The metabolic role of astrocytes in brain has been reviewed in our previous work [60]. Mechanisms of how glucose enters into the astrocytes and neurons are well documented [61]. From the scheme (Figure 2), the presynaptic neuron senses glucose mediated by the T1R2+T1R3 and GLUT2. While the mechanisms, by which the neuron senses glucose through GLUT2, have increasingly been defined, the glucosensing mechanisms of the sweet taste receptor are yet to be understood. It is quite possible that sweet taste receptor can modulate the plasma membrane GLUT2 glucosensor. Functioning cooperatively with GLUT2 to sense the metabolic rate of the intracellular milieu is the G-protein coupled receptor, the inwardly rectifying ATP-dependent potassium channel (K_ATP_ channel) [62, 63]. Transport activity of GLUT2 may be affected through multiple signaling pathways, such as those involving the regulation of GLUT2 and K_ATP_ channel activity. While in Ren et al. [24] study, the signaling activity of GLUT2 was not assessed, their results showed that the inhibition of sweet taste receptor resulted in increase in taste receptor gene expression, suggesting that sweet taste receptors persistently code information about the extracellular glucose level to intracellular milieu, and this might, probably, involve intracellular metabolic sensors, mediating neural activity, gene expression, and membrane receptor trafficking. Although, the mechanisms of the T1R2+T1R3/GLUT2-cooperativity/associativity (or intracellular metabolic sensors) interaction are not known, it can be proposed that T1R2+T1R3 could modulate GLUT2 transport activity through mechanisms as yet unknown. Mechanism of downstream signaling of the neuronal T1R2+T1R3 receptor is similar to that in other cells (Figure 1). The downstream signaling of these receptors can result in changes in extracellular calcium concentration as well as changes in peptide secretions. These biomolecules are sensed by their corresponding receptors in the adjacent neurons/astrocytes, which couple the received information into intracellular signal and cellular activity. The waves of calcium ions, peptide-dependent signaling, can affect collateral neurons and astrocytes by activity dependent signaling and changes in ion waves and regulate gene expression and protein synthesis. Several transcription factors and memory relation genes are activated/deactivated. Intercellular signaling through connexons and pannexons in these cells can modulate their activity.

## Future Direction

Since sweet taste receptor heterodimer, T1R2+T1R3, is a novel glucosensor in the hypothalamus, further research in unraveling the signaling mechanisms associated with this receptor and other glucosensors in key brain areas is necessary to further understanding the association between glucosensors (including their relationship to metabolism) and cognitive functioning. In line with our earlier model of glucose memory facilitation [50, 61, 154], investigation into the mechanisms of neuroastroglial metabolic cooperativity in light of recent discoveries about sweet taste signaling is without doubt necessary.
